# Earthquake Exposure and PTSD Symptoms Among Disaster-Exposed Adolescents: A Moderated Mediation Model of Sleep Problems and Resilience

**DOI:** 10.3389/fpsyt.2021.577328

**Published:** 2021-04-13

**Authors:** Shuo Wang, Xuliang Shi, Xiaoyan Chen, Ya Zhu, Huilin Chen, Fang Fan

**Affiliations:** ^1^College of Education, Hebei University, Baoding, China; ^2^School of Psychology, South China Normal University, Guangzhou, China; ^3^Center for Mental Health Education and Counseling, Guangdong University of Science and Technology, Dongguan, China; ^4^Department of Psychology, University of Bath, Bath, United Kingdom

**Keywords:** earthquake exposure, PTSD symptoms, sleep problems, resilience, adolescents

## Abstract

Considerable studies have explored the potential mechanisms between trauma exposure and PTSD, but little is known about the role of sleep problems and resilience in this relationship. To address this research gap, the present study examined whether sleep problems mediated the relationship between earthquake exposure and PTSD symptoms, and whether this mediating process was moderated by resilience. A sample of 1,342 adolescents (*M*_age_ = 15.54 years, *SD* = 1.26) completed questionnaires regarding earthquake exposure, sleep problems, resilience, and PTSD symptoms 12 months after a devastating earthquake in China. After controlling for demographic variables, earthquake exposure was significantly, and positively associated with PTSD symptoms, and sleep problems partially mediated this relationship. Tests of moderated mediation further revealed that resilience moderated the relationship between earthquake exposure and PTSD symptoms as well as sleep problems and PTSD symptoms. Specifically, the relationship between earthquake exposure and PTSD symptoms was only significant for adolescents with a lower level of resilience; meanwhile, the positive relationship between sleep problems and PTSD symptoms was stronger among low-resilient adolescents. Therefore, sleep-targeted and resilience-based interventions may be effective in alleviating PTSD symptoms resulted from the earthquake.

## Introduction

As is well-known, earthquakes are one of the most common natural disasters, which can not only cause devastating losses of human lives and property damages, but also have long-lasting influences on mental health, especially among children, and teenagers (1–4). Posttraumatic stress disorder (PTSD), characterized by persistent, intrusive memories of the traumatic event, hyperarousal, avoidance of trauma-related cues, and negative changes in thinking and mood (5), is one of the most prevalent mental disorders following the earthquake. For instance, in a meta-analysis of PTSD rates, researchers combined the results of 46 eligible studies, including 76,101 earthquake survivors, and found that the combined incidence of PTSD symptoms after earthquakes was 23.66% (1). Accumulating evidence has suggested that chronic PTSD can lead to adverse consequences, such as substance abuse (6), social withdrawal or loneliness (7), and suicidality (8). Therefore, it is of great importance to pay attention to the influencing factors of PTSD in children and adolescents after the earthquake so as to maintain and even improve their physical and mental health.

Undoubtedly, disaster experiences have been found to be one of the major factors affecting posttraumatic reactions. Some studies have evidenced the “dose-response effect” of trauma severity on PTSD (9–11). That is, the severity of exposure is positively associated with the risk of subsequent PTSD. Regarding objective exposure, studies have found that death or injury of family members, house damage, and property loss are all important predictors for PTSD severity (12, 13). Similarly, individuals' subjective experiences, including perceived life threats and worries about others, also play an important role in explaining PTSD (9, 14). Despite accumulating evidence has confirmed the effect of traumatic exposure on PTSD, little is known about the role of sleep problems and resilience in this relationship. In order to better provide prevention and intervention strategies and reduce the risk of PTSD in adolescents, the present study explored a moderated mediation model to uncover the possible mechanisms underlying this relationship.

Sleep problems, such as insomnia, short sleep time, nightmares, restless sleep, and daytime fatigue, are common in children and adolescents following the disasters (15–17). Three years after the 2013 Ya'an earthquake, Tang et al. investigated the prevalence of sleep problems among 6,132 adolescent survivors, and they found 23.1% of adolescents sleeping <7 h per night, 32.5% having difficulty initiating sleep, 24.2% having difficulty remaining asleep, and 25.3% reporting poor sleep quality (16). Moreover, another study surveyed 1919 junior high school students after the 2011 Japanese earthquake and tsunami, and found that sleep duration was significantly shorter than before the disaster (17). Traumatic events can significantly disrupt individuals' sleep integrity and continuity, and have been shown to be one of the most important precipitants of sleep problems (16, 18, 19). For example, Tang et al. found that both objective and subjective elements of trauma exposure were significantly related to the severity of sleep problems even after adjusting for PTSD symptoms (16). Similarly, using the objective measurement of sleep problems, such as actigraphy and polysomnography, significant abnormalities in sleep parameters were also found in trauma survivors (18, 20).

Sleep problems including insomnia and nightmare are criteria to diagnose PTSD and even be the core components of PTSD (21). Sleep-wake system is among the most vulnerable of regulatory mechanisms following traumatic events (22). Traumatized survivors routinely exhibit marked sleep disruption, especially nocturnal awakenings or hyperarousal, which have been hypothesized to be a pivotal mechanism linking sleep problems and PTSD (19). In addition, sleep can process and store memories of affective experiences while decreasing the emotional charge of memories. When sleep is disturbed, this mechanism cannot function properly, resulting in over-consolidation of emotional component of memories. The dysfunction may increase the susceptibility to developing PTSD (23). Extant research has also supported the notion that sleep problems can predict PTSD symptoms over time (24–27). For example, Wright et al. conducted a prospective assessment of 659 active soldiers at 4 and 8 months after their return from Iraq, and found that insomnia at 4 months significantly contributed to the development and maintenance of PTSD (24). Similarly, another study found that sleep disturbance at 8 weeks postpartum predicted PTSD symptoms 2 years later even after controlling for baseline symptoms (25). In addition, based on polysomnographic data in PTSD, researchers have found that REM fragmentation in the acute posttraumatic period may be an important predictor of PTSD development (27). In summary, sleep problems following trauma may be an important precursor for the subsequent development of psychopathology, including PTSD. Based on the literature reviewed above, it is reasonable to expect that earthquake exposure would be positively related to sleep problems, and the latter would, in turn, be associated with PTSD symptoms. Thus, in this study, we proposed the first hypothesis:

**Hypothesis 1**. Sleep problems would mediate the association between earthquake exposure and PTSD symptoms among adolescent survivors.

Although traumatic exposure exerts a deleterious effect on PTSD, not all adolescents exposed to traumatic events will suffer from psychosocial difficulties. The diathesis-stress model assumed that individuals with vulnerability characteristics are more likely to display heightened sensitivity to environmental pressures, which will increase various likelihoods for developing psychiatric disorders (28, 29). Thus, it is important to examine the personality traits which can alleviate the negative effect of trauma. Guided by this model, the present study mainly focused on resilience, an ability to adapt and successfully cope with traumatic events or adversity (30). Previous studies have also shown that resilience can effectively reduce the susceptibility to adverse psychological outcomes (e.g., PTSD, depression, and suicide) caused by various types of trauma including childhood maltreatment (31, 32), fire accidents (33), and war-related trauma (34). For example, Ying et al. found that resilience moderated the association between traumatic severity and PTSD symptoms (14). Specifically, the negative effect of trauma on PTSD symptoms was stronger in child survivors with lower levels of resilience. Based on the theory and studies reviewed above, we proposed the second hypothesis:

**Hypothesis 2**. Resilience would moderate the relationship between earthquake exposure and PTSD symptoms and/or sleep problems among adolescent survivors.

In addition, despite limited research, it is reasonable to assume that resilience may moderate the relationship between sleep problems and post-traumatic outcomes. Compared with those with lower levels of resilience, high-resilient individuals often adopt adaptive cognitive emotion regulation strategies, such as positive reappraisal, acceptance, positive distancing, and refocus on planning (35, 36), all of which can help survivors to regulate themselves in the face of sleep problems and other mental or physical illness. For example, Min et al. reported that depression/anxiety interacted with resilience could predict suicidal ideation (37). Similarly, another study found that the association between chronic pain and depressive symptoms was stronger for elderly people with a lower level of resilience (38). However, to date, no study has examined resilience as a protective factor against the effects of sleep problems on the risk for PTSD among traumatized adolescents. Therefore, we proposed the third hypothesis:

**Hypothesis 3**. Resilience would moderate the relationship between sleep problems and PTSD symptoms among adolescent survivors.

## Materials and Methods

### Participants and Procedures

This study was part of Wenchuan Earthquake Adolescent Health Cohort (WEAHC). Participants were recruited from one middle and one high school in Dujiangyan City, one of the most affected cities by the earthquake. The two schools were selected for two reasons: (a) Participants came from different social backgrounds and were considered representative of all students in the district; and (b) the school principals were willing to participate in this study. The 7 and 10th graders were selected to participate for the cohort study so as to follow them for at least 2 years before their graduation. A total of 1,573 students participated in the initial survey (about 6 months after the earthquake), with a response rate of 98.3%. Detailed sampling and assessments can be found in Fan et al. (39, 40). In this study, we used data from the second wave of surveys (12 months after the earthquake). In brief, a total of 1,342 students (*M*_age_ = 15.54, *SD* = 1.26) were followed up at this stage. Some students did not participate mainly due to absence from schools on the day of assessment. Among the 1,342 adolescents, 56.9% were females and 83.0% were the only child in their families. Around 51.0% came from urban areas, 16.6% from town areas and 32.4% from rural areas. In terms of their parents, 63.3% of mothers and 57.2% of fathers had an education level of <9 years.

This study was approved by the Human Research Ethics Committee of South China Normal University. All procedures were carried out in accordance with the approved guidelines. Written informed consent was obtained from both the participating students and their parents. For data collection, self-administered questionnaires were distributed to participants and retrieved upon completion. Participants were informed that they could feel free to withdraw from the study at any time.

### Measures

#### Earthquake Exposure

Severity of earthquake exposure was measured with four items: (1) loss and/or injury of family members; (2) house damage; (3) property loss; and (4) direct witness of tragic scenes. The first item included 5 choices: 1=none of the above; 2=moderate injury of family members; 3= serious injury of family members; 4= disappearance of family members; and 5= deaths of family members. The second and third items were rated on a 5-point scale with 1 representing the lowest level of exposure and 5 representing the highest exposure. The last item was scored as follows: 1= not seeing the disaster scene directly and 2= seeing the disaster scene directly. Item scores were added to create a composite score for earthquake exposure, with higher scores (ranging from 4 to 17) indicating higher levels of exposure. The four items to measure earthquake exposure have been used in previous studies widely (2, 39).

#### Post-traumatic Stress Disorder Symptoms

The Post-Traumatic Disorder Self-Rating Scale (PTSD-SS) (41) was used to measure the PTSD symptoms of adolescents 12 months after the earthquake. The PTSD-SS was developed in accordance with the diagnostic criteria of PTSD described in DSM-IV and the Chinese Classification of Mental Disorders. This scale has been widely used to measure PTSD symptoms in Chinese adolescents and adults (42, 43). It entails 24 items, with each item rated on a 5-point scale, ranging from 1 (not at all) to 5 (extremely severe). Item scores were added up to generate a total score ranging from 24 to 120 and a higher total score indicates more severe PTSD symptoms. In the current study, to avoid overlap with PTSD and sleep problems, we excluded 2 items (item 5, and item 12) that related with sleep problems. The PTSD-SS has a satisfactory test-retest reliability, internal consistency and construct validity in Chinese adolescents (41). In the current study, Cronbach's alpha was 0.95.

#### Sleep Problems

Five items from the Chinese version of the Pittsburgh Sleep Quality Index (PSQI-C) (44) were used to assess sleep problems in adolescents after the earthquake. The five items were: (1) “How many hours of actual sleep did you get at night?” (1= 9 h or above, 2 = 7–9 h, 3 = 5–7 h, 4 = below 5 h); (2) “How often have you had difficulty asleep within a week?” (1=never, 2=less than once a week, 3=1–2 times a week, 4=more than 3 times a week); (3) “How often have you had trouble sleeping because you wake up in the middle of the night or early morning?” (response choices were identical to those for the second item); (4) “How would you rate your sleep quality overall?” (1 = very good, 2 = fairly good, 3 = fairly bad, 4 = very bad); (5) “how much of a problem has it been for you to keep up enthusiasm to get things done?” (1 = never, 2 = occasionally, 3 = sometimes, 4 = always). All of the items were rated on a 4-point scale ranging from 1 to 4, with a higher total score indicating severer sleep problems. PSQI-C has demonstrated good psychometric properties (44). In the current study, Cronbach's alpha of the five items was 0.70.

#### Resilience

The Chinese version of Resilience Scale was used to assess adolescents' psychological characteristics and the abilities to cope effectively with adversity (45, 46). This scale includes 25 items and clusters into two subscales: personal competence (e.g., “I can get through difficult times because I have experienced difficulty before”) and acceptance of self and life (e.g., “It's okay if there are people who don't like me”). Participants were asked to respond on a 7-point Likert scale ranging from 1 (“strongly disagree”) to 7 (“strongly agree”). A higher total score (ranging from 25 to 175) indicates a greater degree of resilience. This scale has been widely used in Chinese adolescents with good psychometric properties (45). In the current study, Cronbach's alpha was 0.91.

#### Covariates

Previous studies have found that some demographical factors, such as gender, age, parental education, and location were associated with PTSD symptoms (1–3, 47). Based on the above findings, we chose these variables as possible covariates in the subsequent analysis. Gender was a dichotomous variable (0=male; 1=female). Age was measured by the respondent's age in years. Parental education level was dummy coded as 0 (≤9 years) and 1 (more than 9 years). The residence was recoded as 1 for urban, 2 for town, and 3 for rural.

### Statistical Analyses

Data was analyzed using IBM SPSS version 25. SPSS macro PROCESS was employed to test all of the models (48). The analyses were conducted in four steps. First, descriptive analysis and correlations of all variables were examined. Harman's single-factor test was used to check for common-method variance (49). Second, a mediation analysis using the PROCESS macro (Model 4) was performed in order to test whether the impact of earthquake exposure on PTSD symptoms was mediated by sleep problems. Then, using the PROCESS macro (Model 1), we tested whether resilience moderated the relationship between earthquake exposure and PTSD symptoms. Finally, we tested the moderated mediation model by using the PROCESS macro (Model 59). Moderated mediation addressed the interaction between earthquake exposure and resilience affecting PTSD symptoms (the residual direct relationship), the interaction between earthquake exposure and resilience affecting sleep problems (the first part of the mediation process), and the interaction between sleep problems and resilience affecting PTSD symptoms (the second part of the mediation process). All continuous variables were standardized, and the interaction terms were calculated from these standardized variables. PROCESS offers 95% bias-corrected bootstrap confidence intervals for the indirect effects from 5,000 resamples. Confidence intervals, which do not include zero, were indicators of potential significance. Gender, age, paternal education and residence were controlled for in all analyses. All the variables had data value missing at random, with the rates of missing data ranging from 0.25 to 5.41%. Therefore, expectation-maximization (EM) algorithm was applied to handle the missing data in this study.

## Results

### Preliminary Analyses

Descriptive statistics (means and standard deviations) and correlation matrix were shown in Table 1. Specifically, both earthquake exposure (*r* = 0.21, *p* < 0.01) and sleep problems (*r* = 0.48, *p* < 0.01) were positively associated with PTSD symptoms. In addition, resilience was negatively correlated with PTSD symptoms (*r* = −0.32, *p* < 0.01) and sleep problems (*r* = −0.28, *p* < 0.01). Finally, earthquake exposure was negatively correlated with resilience (*r* = −0.06, *p* < 0.05).

**Table 1:** Descriptive statistics and correlations for all variables.

**Variable**	**M**	**SD**	**1**	**2**	**3**	**4**	**5**	**6**	**7**	**8**	**9**	**10**
Gender	0.57	0.50	1.00									
Age	15.54	1.26	−0.02	1.00								
Sibling	0.17	0.38	0.04	0.11^***^	1.00							
Maternal education	0.37	0.48	0.01	−0.17^***^	−0.06^*^	1.00						
Paternal education	0.43	0.49	−0.03	−0.19^***^	−0.07^*^	0.42^***^	1.00					
Location	1.81	0.89	−0.02	0.37^***^	0.11^***^	−0.40^***^	−0.36^***^	1.00				
Earthquake exposure	9.07	2.44	−0.01	0.09^**^	0.04	−0.01	−0.06^*^	0.05	1.00			
Resilience	112.22	24.19	−0.15^***^	−0.05^*^	−0.03	0.001	0.02	−0.03	−0.06^*^	1.00		
Sleep problems	11.49	2.85	0.13^***^	0.27^***^	0.07^*^	−0.07^*^	−0.04	0.12^**^	0.15^***^	−0.28^***^	1.00	
PTSD symptoms	37.56	14.65	0.11^***^	0.12^***^	0.05	−0.03	−0.07^*^	0.08^**^	0.21^***^	−0.32^***^	0.48^***^	1.00

N = 1,342;

* p < 0.05;

** p < 0.01;

*** *p < 0.001*.

Since the data came from a series of self-assessed questionnaires, Harman's single-factor test was used to check for common-method variance. The results revealed that ten factors with eigenvalues >1.0 were extracted from the unrotated factor analysis, and the first factor accounted for 24% of the variance, which suggested that the samples in our study did not have significant common method bias.

### Testing for Mediation Effect

**Figure 1:**
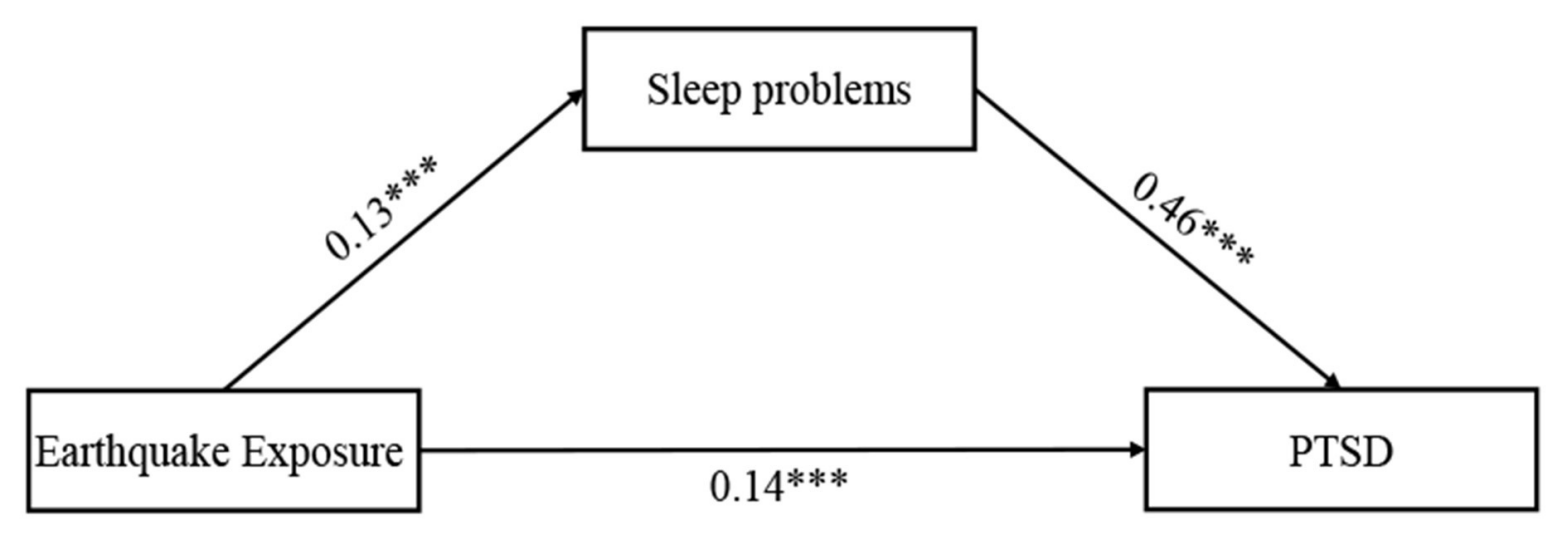
The mediating effect in the association between earthquake exposure and PTSD symptoms.

According to Hypothesis 1, we examined whether sleep problems would mediate the relationship between earthquake exposure and PTSD symptoms (see Figure 1). Before considering the mediating role of sleep problems, we first tested the main effect of earthquake exposure on PTSD symptoms and found that earthquake exposure was positively associated with PTSD symptoms (β = 0.21, *p* < 0.01). Then Model 4 in the PROCESS macro was used to examine the mediating effect. After controlling for adolescents' gender, age, parental education level, and residence, earthquake exposure was positively associated with sleep problems (β = 0.13, *p* < 0.001), which in turn predicted PTSD symptoms (β = 0.46, *p* < 0.001). Bootstrapping analyses further indicated that sleep problems had a significant indirect effect (indirect effect = 0.06, *SE* = 0.01, 95%CI = [0.03, 0.08]). In addition, the residual direct relationship between earthquake exposure and PTSD symptoms was also significant (β = 0.14, *p* < 0.001). Therefore, sleep problems partially mediated the relationship between earthquake exposure and PTSD symptoms. The mediating effect accounted for 29.93% of the total effect.

### Testing for Moderating Effect

**Figure 2:**
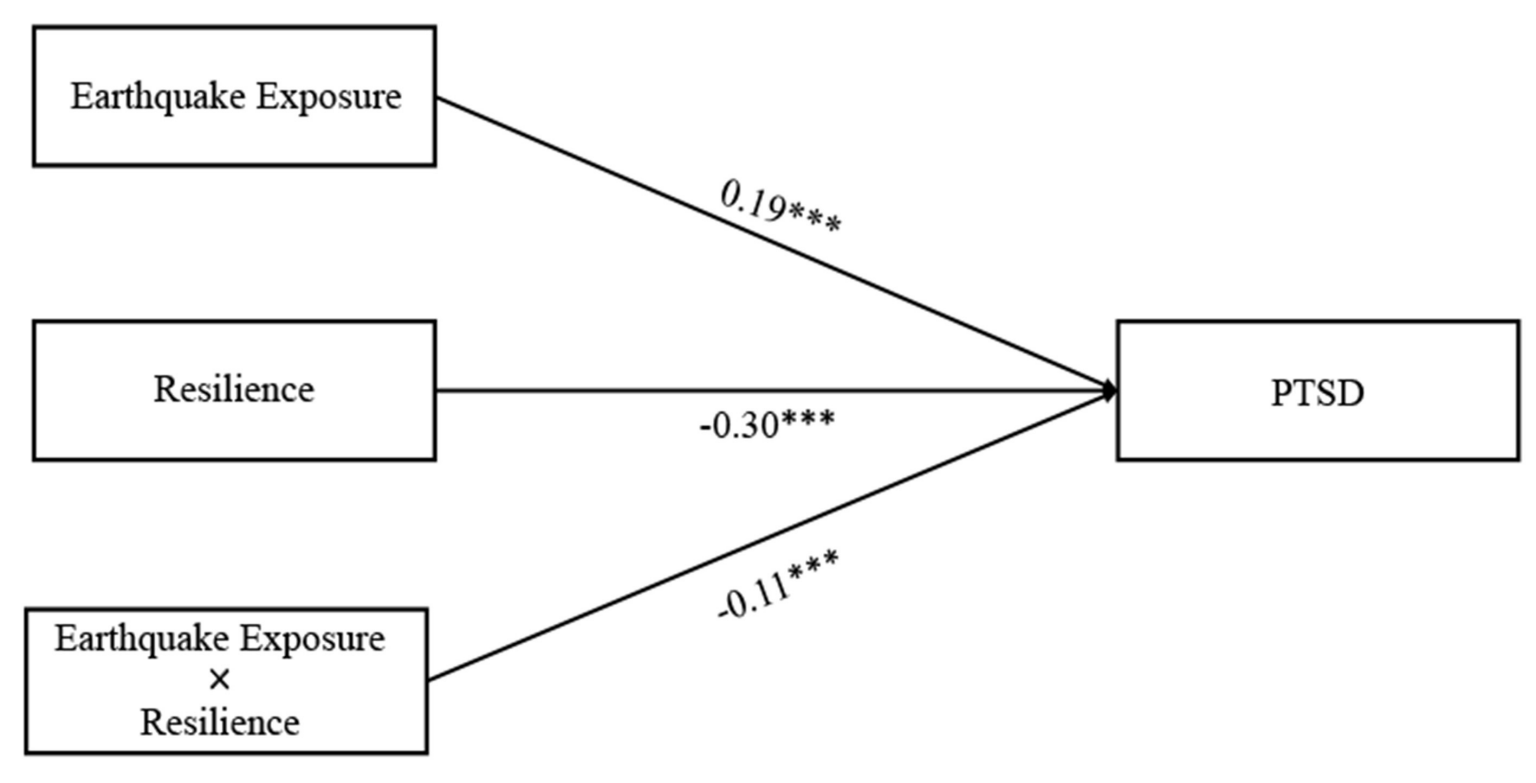
The moderating effect of resilience on the direct relationship between earthquake exposure and PTSD symptoms.

**Figure 3:**
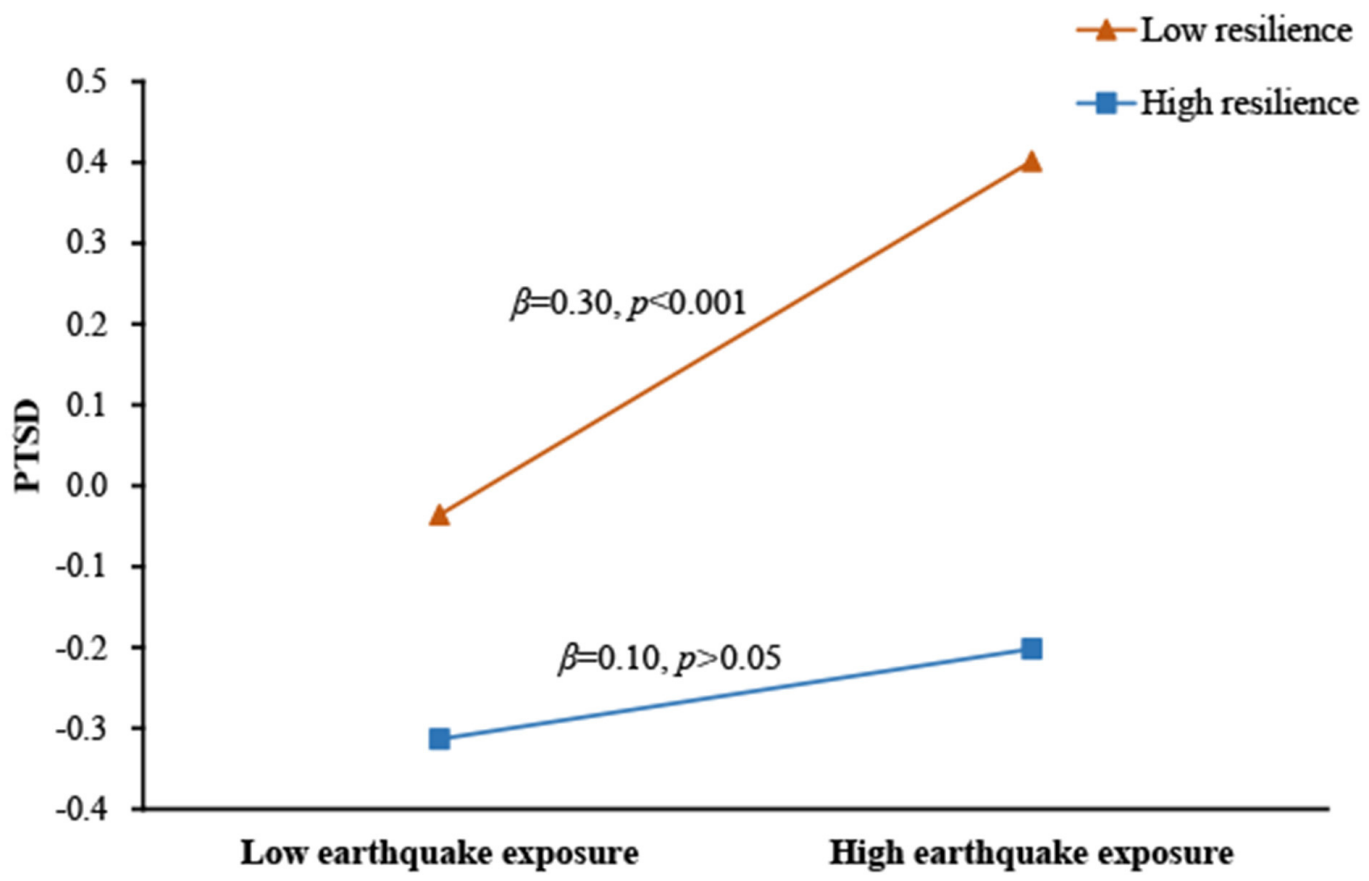
Association between earthquake exposure and PTSD symptoms at higher and lower levels of resilience.

Findings on the interaction between resilience and earthquake exposure as a predictor of PTSD symptoms were presented in Figure 2. Earthquake exposure was positively associated with PTSD symptoms (β = 0.19, *p* < 0.001), whereas resilience was negatively associated with PTSD symptoms (β = −0.30, *p* < 0.001). Moreover, resilience significantly moderated the direct association between earthquake exposure and PTSD symptoms (β = −0.11, *p* < 0.001). For descriptive purposes, we plotted the predicted PTSD symptoms against earthquake exposure, separately for high and low resilience (1 *SD* above the means and 1 *SD* below the means, respectively). The number of people in the high resilience groups was 215 (*M* = 88.03) and the number of people in the low resilience groups was 184 (*M* = 136.41). As shown in Figure 3, a simple slope test revealed that earthquake exposure was significantly and positively associated with PTSD symptoms for adolescents with low resilience (β = 0.30, *p* < 0.001), but the association was not significant for adolescents with high resilience (β = 0.10, *p* > 0.05). Therefore, hypothesis 2 was supported.

### Testing for Moderated Mediation

**Figure 4:**
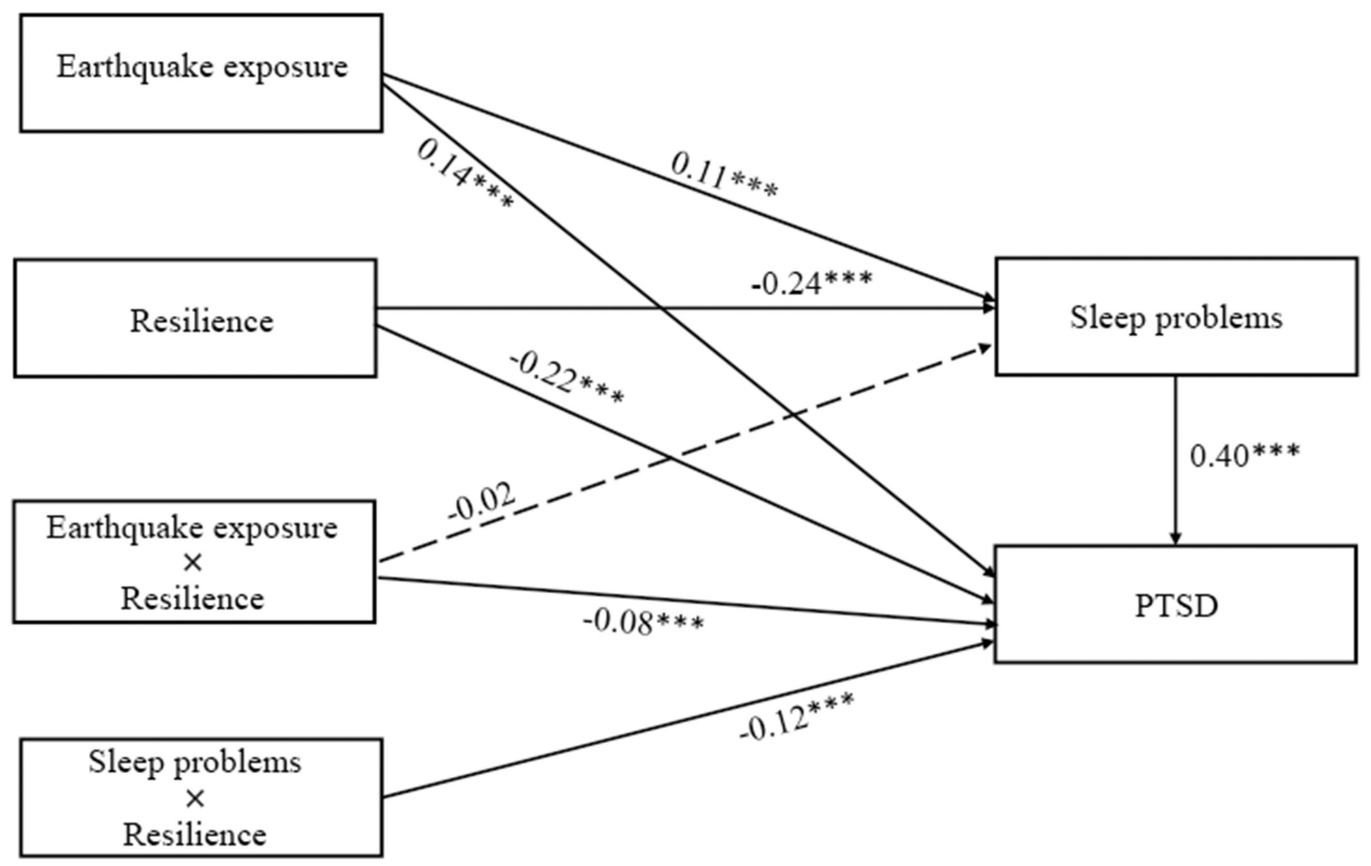
The moderating effect of resilience on the indirect relationship between earthquake exposure and PTSD symptoms.

**Figure 5:**
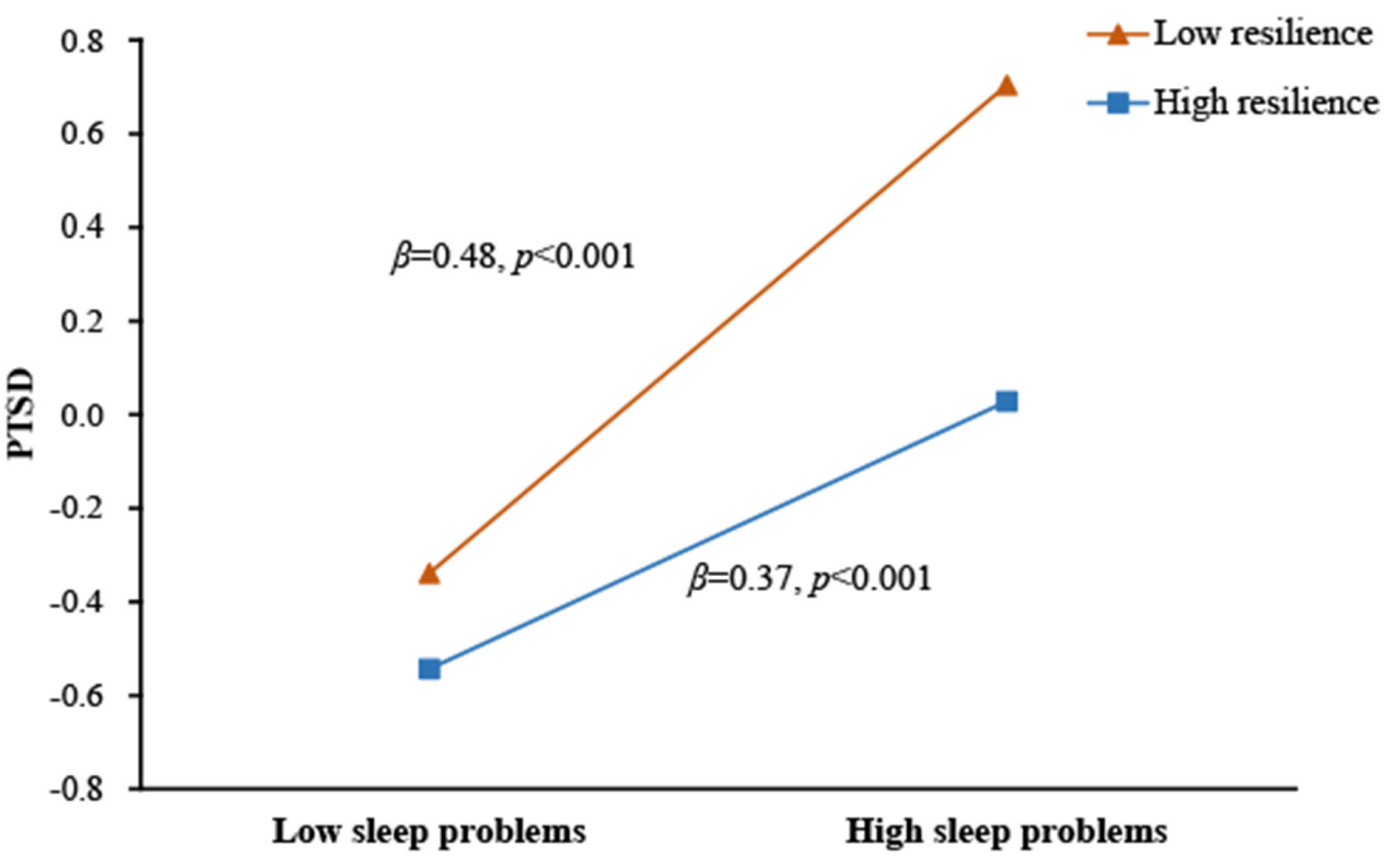
Association between sleep problems and PTSD symptoms at higher and lower levels of resilience.

As specified in Hypothesis 3, we examined the potential moderating role of resilience in the direct and/or indirect associations between earthquake exposure and PTSD symptoms via sleep problems. The results (see Figure 4) indicated that the relationship between sleep problems and PTSD symptoms was significantly moderated by resilience (β = −0.12, *p* < 0.001). Simple slopes analysis (see Figure 5) found that for adolescents with lower levels of resilience (1 *SD* below the mean), higher levels of sleep problems were associated with higher levels of PTSD symptoms (β_simple_ = 0.48, *p* < 0.001). However, for adolescents with higher levels of resilience (1 *SD* above the mean), the effect of sleep problems on PTSD symptoms was weaker (β_simple_ = 0.37, *p* < 0.001). Additionally, although resilience was significantly and negatively associated with sleep problems (β = −0.24, *p* < 0.001), it did not interact with earthquake exposure to predict sleep problems (β = −0.02, *p* > 0.05).

## Discussion

Although evidence suggested that earthquake exposure had a significant impact on PTSD symptoms, few studies have used a process-oriented approach to explore the role of sleep problems and resilience in this relationship. Based on the previous research and theoretical framework, the present study constructed a moderated mediation model to provide a throughout and comprehensive understanding. Our findings indicated that the relationship between earthquake exposure and PTSD symptoms was partially mediated by sleep problems, and this mediating process was moderated by resilience. These findings may be helpful for developing targeted interventions aimed at reducing mental health problems among disaster-exposed adolescents.

Our results showed that sleep problems mediated the association between earthquake exposure and PTSD symptoms. Higher levels of earthquake exposure were associated with more severe sleep problems, which in turn was related with higher levels of PTSD symptoms. Adolescents with high trauma exposure had more sleep-related problems than those with low trauma exposure, which was consistent with previous studies (16, 19, 50). One possible explanation was that trauma-related cues activated the individual's stress/fear response. It was manifested as central and peripheral hyperarousal, contributing to an increase in the level of physiological arousal, which subsequently interfered with sleep onset, continuity, and even led to sleep problems (19). For example, a previous study found that trauma survivors experience significantly increased arousal during sleep, which resulted in a lighter or more fragmented sleep (51). Another tentative explanation was that trauma exposure might increase the risk of substance abuse (52), such as alcohol or drug use, which resulted in sleep problems (53).

Moreover, our findings also showed that sleep problems were positively associated with PTSD symptoms, as evidenced in prior studies (24, 54). Sleep problems themselves may be a stressor, which limit one's capacity to manage stressors, and then potentiate vulnerability to develop mental disorders such as PTSD. It is also possible that sleep problems may weaken individual's daytime coping ability (55) and lead to an increase in avoidance of trauma-related cues, thereby preventing the disappearance of learned fear of trauma-related cues (56). Taken together, trauma-induced sleep problems may be an important risk factor for the development of PTSD. Consistent with previous research (14, 39, 57), resilience was negatively associated with PTSD symptoms. Furthermore, the results supported our hypothesis that resilience was the moderator in the direct relationship between earthquake exposure and PTSD symptoms. More specifically, earthquake exposure was a significant risk factor for PTSD symptoms among adolescents with a lower level of resilience, but not for those with higher resilience. According to the diathesis-stress models of psychopathology, stress will activate individual's diathesis and transform the potential of susceptibility into the presence of psychopathology (28, 29). Resilience as a diathesis and earthquake exposure as a severe stressor will interact to increase the risk of subsequent PTSD. Compared with low-resilient adolescents, high-resilient adolescents would address the stressors adaptively through various ways, such as help-seeking, cognitive restructuring, and emotional expression, all of which would be beneficial for reducing the likelihood of PTSD and preventing psychological breakdowns following traumatic events. These results were consistent with those of previous studies (14, 58). For instance, Fino et al. found that trait resilience moderated effects of war-related trauma on PTSD symptoms, with higher resilience levels buffering the effect of traumatic exposure on PTSD development (58). Also, it is possible that sleep problems and PTSD symptoms were co-occurred (19) or PTSD symptoms predicted sleep problems (59).

As expected, resilience moderated the association between sleep problems and PTSD symptoms. Specially, the detrimental impact of sleep problems on PTSD symptoms was stronger for adolescents with lower resilience vs. higher resilience. When facing stress induced by sleep problems, higher-resilient individuals tend to cope it well. For example, highly resilient adolescents often adopt effective cognitive emotion regulation strategies (60) that serve to attenuate the impact of sleep problems on PTSD. Additionally, resilient adolescents have positive emotions (61) and optimistic attitudes (62), all of which would contribute to reducing the impact of sleep problems on PTSD. In brief, high-resilient people are more positive to face stress induced by sleep problems, and meanwhile deal with the stress effectively, buffering the impact of sleep problems on PTSD.

Contrary to our hypothesis, however, the indirect relation between earthquake exposure and sleep problems was not moderated by resilience. The finding showed that resilience did not protect adolescents from the negative effect of earthquake exposure on sleep problems. In other words, resilience only played a direct rather than a buffer role in this relationship. This finding was consistent with a previous study by Chambers and Belicki (63). They surveyed 97 college students and found that resilient characteristics did not protect trauma and abuse survivors from sleep or dream disturbance. Sleep problems such as insomnia and nightmares are significant characteristics of the human neurobiological and physiological response to trauma (19). Most previous studies have shown that subjects with insomnia have increased secretion of the hypothalamic-pituitary-adrenal (HPA) axis and the sympathetic nervous system (64, 65). Based on these findings, we inferred that resilience could only buffer the adverse effects of traumatic events on psychological pain, such as PTSD and depression, but could not mitigate the adverse effects of traumatic events on physiological symptoms, such as insomnia. Due to the lack of research to examine this topic, large-scale studies are warranted to further confirmed these findings.

Although our data provided new evidence pertaining to the mediating effect of sleep problems and moderating effect of resilience on the relationship between earthquake exposure and PTSD symptoms for disaster-exposed adolescents, a number of limitations should be noted when interpreting these findings. First, PTSD symptoms, such as intrusion, may also predict the development of sleep problems. However, due to the cross-sectional design, it's impossible to verify causality or directionality among these variables. Future studies are warranted to use prospective designs with multiple-wave assessments so as to explore these longitudinal developments. Second, all measures in our study were based on adolescents' self-reports, which may be biased because of the social desirability. Multiple assessments and information sources (e.g., self-report, caregivers' report, and teachers' report) can be employed to enhance the reliability of the results in future studies. Third, only five items were used to measure sleep problems in our study. Although brief measures about sleep problems have already been supported for the validity (54, 66), future research would benefit from other better-structured scales (e.g., Pittsburgh sleep quality index, PSQI). Last but not least, participants in this study were only recruited from adolescents who had been exposed to a devastating earthquake, making it limited to generalize the conclusions to other age samples or various types of trauma (e.g., acute or man-made traumatic events or chronic interpersonal events). Briefly, these findings should be confirmed in future studies with larger and more representative samples in various population ranges.

Despite these limitations, there were several important practical implications. On the one hand, our results suggested that sleep problem was a critical component bridging earthquake exposure and PTSD symptoms. It is noteworthy that sleep problem represents a modifiable risk factor, and people are more willing to receive treatment for sleep-related problems than for other mental disorders. Therefore, early identification and treatment of sleep problems may be a promising strategy for the prevention and intervention of PTSD in response to a disaster. Previous empirical studies have shown that evidence-based psychotherapies, such as mindfulness-based cognitive therapy (MBCT) (67) and cognitive behavioral therapy for insomnia (CBT-I) (68) could not only improve sleep quality, but also alleviate PTSD symptoms in clinical patients. On the other hand, our study found that resilience played a moderating role in the direct and indirect relation between earthquake exposure and PTSD symptoms. The result indicated that interventions targeting individual resilience might be effective for those who have experienced severe trauma or sleep problems. In conclusion, we suggest that mental health professionals in the school settings should develop and offer some resilience-oriented prevention and intervention programs (e.g., communication skill training, mindfulness meditation training, and cognitive reappraisal training), in order to promote and strengthen resilience among disaster-exposed adolescents (36, 69).

In summary, our study constructed a moderated mediation model to examine the relationship between earthquake exposure and PTSD symptoms. Sleep problems served as one potential mediator through which earthquake exposure is related to PTSD symptoms. Moreover, resilience buffered the effects of both earthquake exposure and sleep problems on PTSD symptoms. Our findings highlight the importance of an integrative approach to assessing and improving sleep problems and resilience, which can be a promising strategy for the prevention and intervention of adolescents' PTSD symptoms.

## Data Availability Statement

The raw data supporting the conclusions of this article will be made available by the authors, without undue reservation.

## Ethics Statement

This study was approved by the Human Research Ethics Committee of South China Normal University. All procedures were carried out in accordance with the approved guidelines. Written informed consent was obtained from both the participating students and their parents.

## Author Contributions

SW: data analysis and paper revision. XS: study design, data collection, data analysis, and paper revision. XC, YZ, and HC: paper revision. FF: study design.

## Conflict of Interest

The authors declare that the research was conducted in the absence of any commercial or financial relationships that could be construed as a potential conflict of interest.

## References

[1] Dai W Chen L Lai Z Li Y Wang J Liu A. The incidence of post-traumatic stress disorder among survivors after earthquakes:a systematic review and meta-analysis. BMC Psychiatr. (2016) 16:188. 10.1186/s12888-016-0891-9 27267874 PMC4895994

[2] Fan F Zhang Y Yang Y Mo L Liu X. Symptoms of posttraumatic stress disorder, depression, and anxiety among adolescents following the 2008 Wenchuan earthquake in China. J Trauma Stres. (2011) 24:44–53. 10.1002/jts.20599 21351164

[3] Liang Y Cheng J Ruzek JI Liu Z. Posttraumatic stress disorder following the 2008 Wenchuan earthquake: a 10-year systematic review among highly exposed populations in China. J Affect Disorders. (2019) 243:327–39. 10.1016/j.jad.2018.09.047 30261448

[4] Rubens SL Felix ED Hambrick EP. A meta-analysis of the impact of natural disasters on internalizing and externalizing problems in youth. J Trauma Stres. (2018) 31:332–41. 10.1002/jts.22292 29870078 PMC6055700

[5] American Psychiatric Association. Diagnostic and statistical manual of mental disorders (DSM-5®). American Psychiatric Pub. (2013). 10.1176/appi.books.9780890425596

[6] Kachadourian LK Pilver CE Potenza MN. PTSD, and binge and hazardous drinking among women and men:findings from a national study. J Psychiatr Res. (2014). 55:35–43. 10.1016/j.jpsychires.2014.04.018 24838049 PMC4094352

[7] van der Velden PG Pijnappel B van der Meulen E. Potentially traumatic events have negative and positive effects on loneliness, depending on PTSD-symptom levels:evidence from a population-based prospective comparative study. Soc Psychiatry Psychiatr Epidemiol. (2018) 53:195–206. 10.1007/s00127-017-1476-8 29288318 PMC5816097

[8] Guo J He H Fu M Han Z Qu Z Wang X. Suicidality associated with PTSD. depression, and disaster recovery status among adult survivors 8 years after the 2008 Wenchuan earthquake in China. Psychiatry Res. (2017) 253:383–90. 10.1016/j.psychres.2017.04.022 28437765

[9] Furr JM Comer JS Edmunds JM Kendall PC. Disasters and youth:a meta-analytic examination of posttraumatic stress. J Consult Clin Psychol. (2010) 78:765–80. 10.1037/a0021482 21114340

[10] Johnson H Thompson A. The development and maintenance of post-traumatic stress disorder (PTSD) in civilian adult survivors of war trauma and torture: a review. Clin Psychol Rev. (2008) 28:36–47. 10.1016/j.cpr.2007.01.017 17383783

[11] Kolassa IT Ertl V Eckart C Kolassa S Onyut LP Elbert T. Spontaneous remission from PTSD depends on the number of traumatic event types experienced. Psychol Trauma. (2010) 2:169–74. 10.1037/a0019362

[12] Cao X Wang L Cao C Zhang J Liu P Zhang B. Patterns of DSM-5 posttraumatic stress disorder and depression symptoms in an epidemiological sample of Chinese earthquake survivors:A latent profile analysis. J Affect Disord. (2015) 186:58–65. 10.1016/j.jad.2015.06.058 26231442

[13] Tang W Zhao J Lu Y Zha Y Liu H Sun Y. Suicidality, posttraumatic stress, and depressive reactions after earthquake and maltreatment:a cross-sectional survey of a random sample of 6132 Chinese children and adolescents. J Affect Disorders. (2018) 232:363–9. 10.1016/j.jad.2018.02.081 29510354

[14] Ying L Wu X Lin C Jiang L Traumatic severity and trait resilience as predictors of posttraumatic stress disorder and depressive symptoms among adolescent survivors of the Wenchuan earthquake. PLoS ONE. (2014) 9:89401. 10.1371/journal.pone.0089401 24586751 PMC3935868

[15] Brown TH Mellman TA Alfano CA Weems CF. Sleep fears, sleep disturbance, and PTSD symptoms in minority youth exposed to Hurricane Katrina. J Trauma Stres. (2011). (2011) 24:575–80. 10.1002/jts.20680 21898601

[16] Tang W Lu Y Yang Y Xu J. An epidemiologic study of self-reported sleep problems in a large sample of adolescent earthquake survivors: the effects of age, gender, exposure, and psychopathology. J Psychosomat Res. (2018) 113:22–9. 10.1016/j.jpsychores.2018.07.006 30190044

[17] Iwadare Y Usami M Ushijima H Tanaka T Watanabe K Kodaira M. Changes in traumatic symptoms and sleep habits among junior high school students after the Great East Japan Earthquake and Tsunami. Sleep Biol Rhythms. (2014) 12:53–61. 10.1111/sbr.12047

[18] Brindle RC Cribbet MR Samuelsson LB Gao C Frank E Krafty RT. The relationship between childhood trauma and poor sleep health in adulthood. Psychosom Med. (2018) 80:200–7. 10.1097/PSY.0000000000000542 29215455 PMC5794533

[19] Sinha SS. Trauma-induced insomnia: a novel model for trauma and sleep research. Sleep Med Rev. (2016) 25:74–83. 10.1016/j.smrv.2015.01.008 26140870

[20] Mellman TA David D Kulick-Bell R Hebding J Nolan B. Sleep disturbance and its relationship to psychiatric morbidity after Hurricane Andrew. Am J Psychiat. (1995) 152:1659–63. 10.1176/ajp.152.11.1659 7485631

[21] Spoormaker VI Montgomery P. Disturbed sleep in post-traumatic stress disorder: secondary symptom or core feature? Sleep Med Rev. (2008) 12:169–84. 10.1016/j.smrv.2007.08.008 18424196

[22] Sadeh A. Stress, trauma, and sleep in children. Child Adol Psych. (1996) 5:685–700. 10.1016/S1056-4993(18)30356-0

[23] Walker M van der Helm E. Overnight therapy? The role of sleep in emotional brain processing. Psychol Bull. (2009) 135:731–48. 10.1037/a0016570 19702380 PMC2890316

[24] Wright KM Britt TW Bliese PD Adler AB Picchioni D Moore D. Insomnia as predictor versus outcome of PTSD and depression among Iraq combat veterans. J Clin Psychol. (2011) 67:1240–58. 10.1002/jclp.20845 22065464

[25] Gathus-Niegel S Ayers S von Soest T Torgersen L Eberhard-Gran M. Maintaining factors of posttraumatic stress symptoms following childbirth: a population-based, two-year follow-up study. J Affect Disord. (2015) 172:146–52. 10.1016/j.jad.2014.10.003 25451409

[26] Pigeon WR Campbell CE Possemato K and Ouimette P. Longitudinal relationships of insomnia, nightmares, and PTSD severity in recent combat veterans. J Psychosom Res. (2013) 75:546–50. 10.1016/j.jpsychores.2013.09.004 24290044

[27] Mellman TA Bustamante V Fins AI Pigeon WR Nolan B. REM sleep and the early development of posttraumatic stress disorder. Am J Psychiat. (2002) 159:1696–701. 10.1176/appi.ajp.159.10.1696 12359675

[28] McKeever VM Huff ME. A diathesis-stress model of posttraumatic stress disorder:Ecological, biological, and residual stress pathways. Rev Gen Psychol. (2003) 7:237–50. 10.1037/1089-2680.7.3.237

[29] Monroe SM Simons AD. Diathesis-stress theories in the context of life stress research:implications for the depressive disorders. Psychol Bull. (1991) 110:406–25. 10.1037/0033-2909.110.3.406 1758917

[30] Bonanno Loss GA Trauma and human resilience: have we underestimated the human capacity to thrive after extremely aversive events? Am Psychol. (2004) 59:20–8. 10.1037/0003-066X.59.1.20 14736317

[31] Poole JC Dobson KS Pusch D. Childhood adversity and adult depression: the protective role of psychological resilience. Child Abuse Negl. (2017) 64:89–100. 10.1016/j.chiabu.2016.12.012 28056359

[32] Roy A Carli V Sarchiapone M. Resilience mitigates the suicide risk associated with childhood trauma. J Affect Disord. (2011) 13:591–4. 10.1016/j.jad.2011.05.006 21621850

[33] Lee JS Ahn YS Jeong KS Chae JH Choi KS. Resilience buffers the impact of traumatic events on the development of PTSD symptoms in firefighters. J Affect Disorders. (2014) 162:128–33. 10.1016/j.jad.2014.02.031 24767017

[34] Pietrzak RH Johnson DC Goldstein MB Malley JC Rivers AJ Morgan CA. Psychosocial buffers of traumatic stress, depressive symptoms, and psychosocial difficulties in veterans of Operations Enduring Freedom and Iraqi Freedom:the role of resilience, unit support, and postdeployment social support. J Affect Disord. (2010) 120:188–92. 10.1016/j.jad.2009.04.015 19443043

[35] Min JA Yu JJ Lee CU Chae JH. Cognitive emotion regulation strategies contributing to resilience in patients with depression and/or anxiety disorders. Compr Psychiat. (2013) 54:1190–7. 10.1016/j.comppsych.2013.05.008 23806709

[36] Southwick SM Charney DS. The science of resilience:implications for the prevention and treatment of depression. Science. (2012) 338:79–82. 10.1126/science.1222942 23042887

[37] Min JA Lee CU Chae JH. Resilience moderates the risk of depression and anxiety symptoms on suicidal ideation in patients with depression and/or anxiety disorders. Compr Psychiat. (2015) 56:103–11. 10.1016/j.comppsych.2014.07.022 25248467

[38] Bauer H Emeny R Baumert J Ladwig KH. Resilience moderates the association between chronic pain and depressive symptoms in the elderly. Eur J Pain. (2016) 20:1253–65. 10.1002/ejp.850 26914727

[39] Fan F Long K Zhou Y Zheng Y Liu X. Longitudinal trajectories of post-traumatic stress disorder symptoms among adolescents after the Wenchuan earthquake in China. Psychol Med. (2015) 45:2885–96. 10.1017/S0033291715000884 25990926

[40] Fan F Zhou Y Mo L Zhang W Xie J Liu X. Cohort profile:the Wenchuan earthquake adolescent health cohort study. Int J Epidemiol. (2017) 46:27–8. 10.1093/ije/dyw013 27044503

[41] Liu X Ma D Liu L Zhao G Li C Yang J. Development of the post-traumatic stress disorder self-rating scale and its reliability and validity. Chinese J Behav Med Sci. (1998) 7:93–96.

[42] Liu Q Jiang M Yang Y Zhou H Zhou Y Yang M. Prevalence of posttraumatic stress disorder (PTSD) and its correlates among junior high school students at 53 months after experiencing an earthquake. Dis Med Public Health Prep. (2019) 13:414–8. 10.1017/dmp.2018.76 30207265

[43] Wang B Ni C Chen J Liu X Wang A Shao Z. Posttraumatic stress disorder 1 month after 2008 earthquake in China:Wenchuan earthquake survey. Psychiatry Res. (2011) 187:392–6. 10.1016/j.psychres.2009.07.001 20537713

[44] Liu X Tang M Hu L Wang A Wu H Zhao G. Reliability and validity of the Pittsburgh sleep quality index. Chinese J Psychiatr. (1996) 29:103–7.28455545

[45] Lei M Li C Xiao X Qiu J Dai Y Zhang Q. Evaluation of the psychometric properties of the Chinese version of the Resilience Scale in Wenchuan earthquake survivors. Compr Psychiat. (2012) 53:616–22. 10.1016/j.comppsych.2011.08.007 22001021

[46] Wagnild GM Young HM. Development and psychometric evaluation of the Resilience Scale. J Nurs Measurement. (1993) 1:165–78. 7850498

[47] Trickey D Siddaway AP Meiser-Stedman R Serpell L Field AP. A meta-analysis of risk factors for post-traumatic stress disorder in children and adolescents. Clin Psychol Rev. (2012) 32:122–38. 10.1016/j.cpr.2011.12.001 22245560

[48] Hayes AF. Introduction to Mediation, Moderation, and Conditional Process Analysis: A Regression-Based Approach. Ney York, NY: Guilford Publications (2017).

[49] Podsakoff PM Organ DW. Self-reports in organizational research:Problems and prospects. J Manag. (1986) 12:531–44. 10.1177/0149206386012004088452065

[50] Kliewer W Lepore SJ. Exposure to violence, social cognitive processing, and sleep problems in urban adolescents. J Youth Adolesc. (2015) 44:507–17. 10.1007/s10964-014-0184-x 25218396 PMC4294953

[51] Pillar G Malhotra A Lavie P. Post-traumatic stress disorder and sleep-what a nightmare! Sleep Med Rev. (2000) 4:183–200. 10.1053/smrv.1999.0095 12531165

[52] Weiss NH Bold KW Contractor AA Sullivan TP Armeli S Tennen H. Trauma exposure and heavy drinking and drug use among college students: identifying the roles of negative and positive affect lability in a daily diary study. Addict Behav. (2018) 79:131–7. 10.1016/j.addbeh.2017.12.015 29289852 PMC5895102

[53] Ogeil RP Cheetham A Mooney A Allen NB Schwartz O Byrne ML. Early adolescent drinking and cannabis use predicts later sleep-quality problems. Psychol Addict Behav. (2019) 33:266–73. 10.1037/adb0000453 30869923

[54] Fan F Zhou Y Liu X. Sleep disturbance predicts posttraumatic stress disorder and depressive symptoms: a cohort study of Chinese adolescents. J Clin Psychiatry. (2017) 78:882–8. 10.4088/JCP.15m10206 27574834

[55] Short MA Gradisar M Lack LC Wright HR Dohnt H. The sleep patterns and well-being of Australian adolescents. J Adolesc. (2013) 36:103–110. 10.1016/j.adolescence.2012.09.008 23088812

[56] Rothbaum BO Mellman TA. Dreams and exposure therapy in PTSD. J Trauma Stres. (2001) 14:481–90. 10.1023/A:1011104521887 11534880

[57] Zhou X Wu X An Y. Understanding the relationship between trauma exposure and depression among adolescents after earthquake: the roles of fear and resilience. Front Psychol. (2016) 7:2044. 10.3389/fpsyg.2016.02044 28082947 PMC5183574

[58] Fino E Mema D Russo P. War trauma exposed refugees and posttraumatic stress disorder: the moderating role of trait resilience. J Psychosomat Res. (2020) 129:109905. 10.1016/j.jpsychores.2019.109905 31869693

[59] Zhou X Wu X Chen Q Zhen R. Why did adolescents have sleep problems after earthquakes? Understanding the role of traumatic exposure, fear, and PTSD. Scand J Psychol. (2017) 58:221–7. 10.1111/sjop.12366 28543322

[60] Kay SA. Emotion regulation resilience:overlooked connections. Ind Organ Psychol. (2016) 9:411–5. 10.1017/iop.2016.31

[61] Tugade MM Fredrickson BL. Resilient individuals use positive emotions to bounce back from negative emotional experiences. J Pers Soc Psychol. (2004) 86:320–33. 10.1037/0022-3514.86.2.320 14769087 PMC3132556

[62] Riolli L Savicki V Cepani A. Resilience in the face of catastrophe: optimism, personality, and coping in the Kosovo crisis. J Appl Soc Psychol. (2002) 32:1604–27. 10.1111/j.1559-1816.2002.tb02765.x

[63] Chambers E Belicki K. Using sleep dysfunction to explore the nature of resilience in adult survivors of childhood abuse or trauma. Child Abuse Negl. (1998) 22:753–8. 10.1016/S0145-2134(98)00059-3 9717612

[64] Vgontzas A Bixler E Lin H Prolo P Mastorakos G Vela-Bueno A. Chronic insomnia is associated with nyctohemeral activation of the hypothalamic-pituitary-adrenal axis:clinical implications. J Clin Endocrinol Metab. (2001) 86:3787–94. 10.1210/jcem.86.8.7778 11502812

[65] Basta M Chrousos GP Vela-Bueno A Vgontzas AN. Chronic insomnia and the stress system. Sleep Med Clin. (2007) 2:279–91. 10.1016/j.jsmc.2007.04.002 18071579 PMC2128619

[66] Zhang L Yang Y Liu Z Jia C and Liu X. Sleep disturbance mediates the association between intrafamily conflict and mental health problems in Chinese adolescents. Sleep Med. (2018) 46:74–80. 10.1016/j.sleep.2018.02.012 29773215

[67] Nakamura Y Lipschitz DL Landward R Kuhn R West G. Two sessions of sleep-focused mind–body bridging improve self-reported symptoms of sleep and PTSD in veterans: a pilot randomized controlled trial. J Psychosomat Res. (2011) 70:335–45. 10.1016/j.jpsychores.2010.09.007 21414453

[68] Lamarche LJ De Koninck J. Sleep disturbance in adults with posttraumatic stress disorder: a review. J Clin Psychiatr. (2007) 68:1257–70. 10.4088/JCP.v68n0813 17854251

[69] Dray J Bowman J Campbell E Freund M Wolfenden L Hodder RK. Systematic review of universal resilience-focused interventions targeting child and adolescent mental health in the school setting. J Am Acad Child Adolesc Psychiatr. (2017) 56:813–24. 10.1016/j.jaac.2017.07.780 28942803

